# Phosphate effect on filipin production and morphological differentiation in *Streptomyces filipinensis* and the role of the PhoP transcription factor

**DOI:** 10.1371/journal.pone.0208278

**Published:** 2018-12-06

**Authors:** Eva G. Barreales, Tamara D. Payero, Antonio de Pedro, Jesús F. Aparicio

**Affiliations:** Area de Microbiología, Departamento de Biología Molecular, Universidad de León, León, Spain; Universite Paris-Sud, FRANCE

## Abstract

The biosynthesis of the antifungal filipin in *Streptomyces filipinensis* is very sensitive to phosphate regulation. Concentrations as low as 2.5 mM block filipin production. This effect is, at least in part, produced by repression of the transcription of most filipin biosynthetic genes. The role of the two-component PhoRP system in this process was investigated. The *phoRP* system of *S*. *filipinensis* was cloned and transcriptionally characterised. PhoP binds to two PHO boxes present in one of its two promoters. Filipin production was greatly increased in Δ*phoP* and Δ*phoRP* mutants, in agreement with a higher transcription of the *fil* genes, and the effect of phosphate repression on the antibiotic production of these strains was significantly reduced. No PhoP binding was observed by electrophoretic mobility gel shift assays (EMSAs) with the promoter regions of the *fil* gene cluster thus suggesting an indirect effect of mutations. Binding assays with cell-free extracts from the wild-type and mutant strains on *fil* genes promoters revealed retardation bands in the parental strain that were absent in the mutants, thus suggesting that binding of the putative transcriptional regulator or regulators controlled by PhoP was PhoP dependent. Noteworthy, PhoP or PhoRP deletion also produced a dramatic decrease in sporulation ability, thus indicating a clear relationship between the phosphate starvation response mediated by PhoP and the sporulation process in *S*. *filipinensis*. This effect was overcome upon gene complementation, but also by phosphate addition, thus suggesting that alternative pathways take control in the absence of PhoRP.

## Introduction

Soil-dwelling streptomycetes undergo a complex life cycle with morphological differentiation and sporulation. These bacteria are well-known for their ability to produce a great variety of secondary metabolites [1]. These chemically diverse molecules have found multiple applications, particularly in medicine and in agriculture, and include antibacterial antibiotics, antitumor agents, immunosuppressants, anthelmintic agents, and fungicides [2]. Filipin III is one of those fungicide agents, a 28-membered macrolactone pentaene produced by *Streptomyces filipinensis* and other *Streptomyces* strains [3–6]. This compound has a potent antifungal activity, derived from its interaction with the main sterol of fungal membranes ergosterol which disrupts the plasma membrane and leads to fungal death [7,8]. Unlike most polyenes macrolides, filipin III has also high affinity for cholesterol [9], and therefore can interact with the cell membranes of mammals. This is one of the reasons why filipin III is widely used as a tool for the diagnosis of Niemann-Pick type C disease, a characteristic cholesterol storage disorder of genetic origin [10], and for the detection and the quantitation of cholesterol in biological membranes [11]. Unlike most polyene macrolides, filipin is devoid of sugar, constituting the archetype of non-glycosylated polyenes, and this is the reason, together with the lack of a charged carboxyl group in its structure, why filipin interacts with sterols differently to glycosylated polyenes forming complexes inside the membrane bilayer [12]. Being a polyene, it is synthesized by type I polyketide synthases. Its biosynthetic gene cluster (*pte*) was first identified in the avermectin producing *S*. *avermitilis* NRRL 8165 upon sequencing of its genome [13] and some of its genes have been characterised [14–17]. Recently, a highly similar biosynthetic gene cluster (*fil*) has been cloned and characterised in the industrial filipin producer *S*. *filipinensis*, finding one extra PadR-like encoding regulatory gene (*filI*) involved in antifungal biosynthesis [18].

Production of secondary metabolites by *Streptomyces* is regulated in response to nutritional status alteration, and occurs in a growth-phase dependent manner, being governed by complex intertwined regulatory networks that respond to environmental and physiological signals [19–21]. The onset of secondary metabolite biosynthesis generally occurs at the end of rapid vegetative growth and is often provoked by nutritional limitations. One of this key stress-producing factors is phosphate starvation. Inorganic phosphate (Pi) is essential for life yet it is usually scarce in nature, thus bacteria have developed strategies to adapt to this condition. In *Streptomyces*, the best studied regulatory system involved in the sensing and response to phosphate starvation is the highly conserved two-component signal transduction system PhoRP [22], although other systems have been reported [23]. PhoR is a membrane sensor histidine kinase that upon phosphate deprivation phosphorylates its cognate cytoplasmic response regulator PhoP at an aspartic residue, which then binds to specific sequences in target promoters (so called PHO boxes), activating or repressing their transcription [24]. These PHO boxes are formed by direct repeat units (DRus) of 11 nucleotides composed by seven well conserved and four less conserved nucleotides, being GTTCACC the most conserved motif in *S*. *coelicolor* [25]. Members of the *pho* regulon activated by PhoP include genes encoding extracellular hydrolytic enzymes that allow cell to obtain Pi from organic sources, its transport, and enzymes involved in its storage, and genes for cell wall biosynthesis, but PhoP also represses a number of genes involved in nitrogen assimilation, oxidative phosphorylation, nucleotide biosynthesis and glycogen metabolism [26–28], thus constituting a “master” regulator in this model actinomycete. Members of the *pho* regulon have recently been identified in other species such as *S*. *avermitilis* or *S*. *tsukubaensis* [29,30].

In *Streptomyces* genomes, the *phoR* and *phoP* genes form an operon that is self-activated by PhoP binding to its promoter [24]. Divergently situated there is a gene, *phoU*, that modulates the signal transduction cascade [31,32]. In *S*. *lividans*, PhoP can also be transcribed from a promoter located in the 3'-region of *phoR* [31]. The effect of *phoP* deletion on antibiotic production has only been determined in five *Streptomyces* strains. *S*. *lividans* overproduces actinorhodin and undecylprodigiosin in complex media upon gene deletion [22]. Similar results have been observed with pimaricin in *S*. *natalensis* and *S*. *lydicus* [33,34], or with avermectin in *S*. *avermitilis* [35], but in *S*. *coelicolor* not only there is no overproduction of actinorhodin and undecylprodigiosin [36] but their production is repressed in defined media [37].

In order to deepen our knowledge about the mechanism of phosphate control of filipin biosynthesis, it was of great interest to study transcriptional control of the filipin genes by phosphate, and the regulatory role of PhoP on filipin production in *S*. *filipinensis*.

## Results and discussion

### Effect of phosphate on filipin production

**Fig 1:**
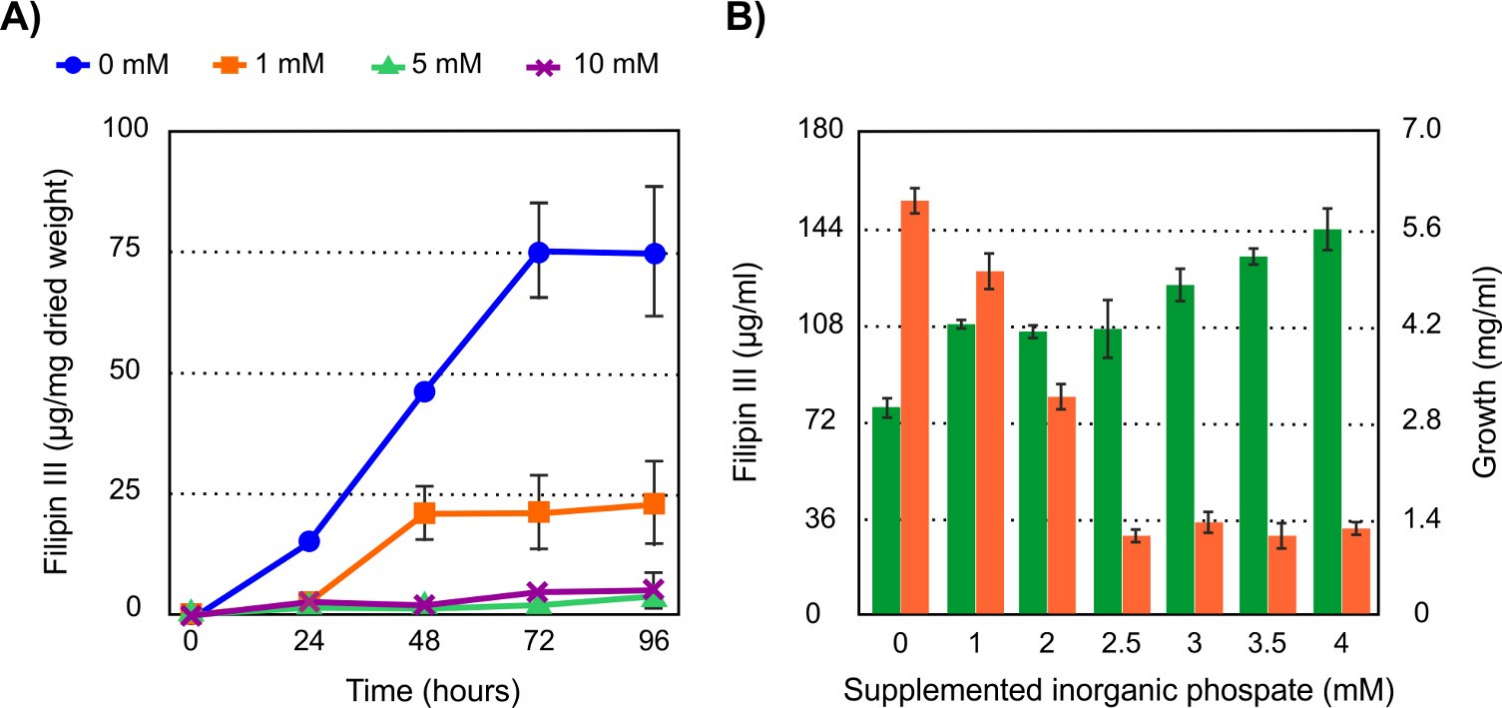
Phosphate effect on filipin III production. A) Effect of increasing inorganic phosphate concentrations (0, 1, 5, and 10 mM) on the specific production of filipin (expressed as μg of filipin per mg of dry weight) by the wild type *S*. *filipinensis* DSM 40112 in YEME medium. B) Determination of the threshold for saturation of phosphate depressive effect on filipin biosynthesis. Volumetric production of filipin is indicated in orange and growth in green. Data are the average of three duplicate flasks. Vertical bars indicate standard deviation of the mean values.

**Fig 2:**
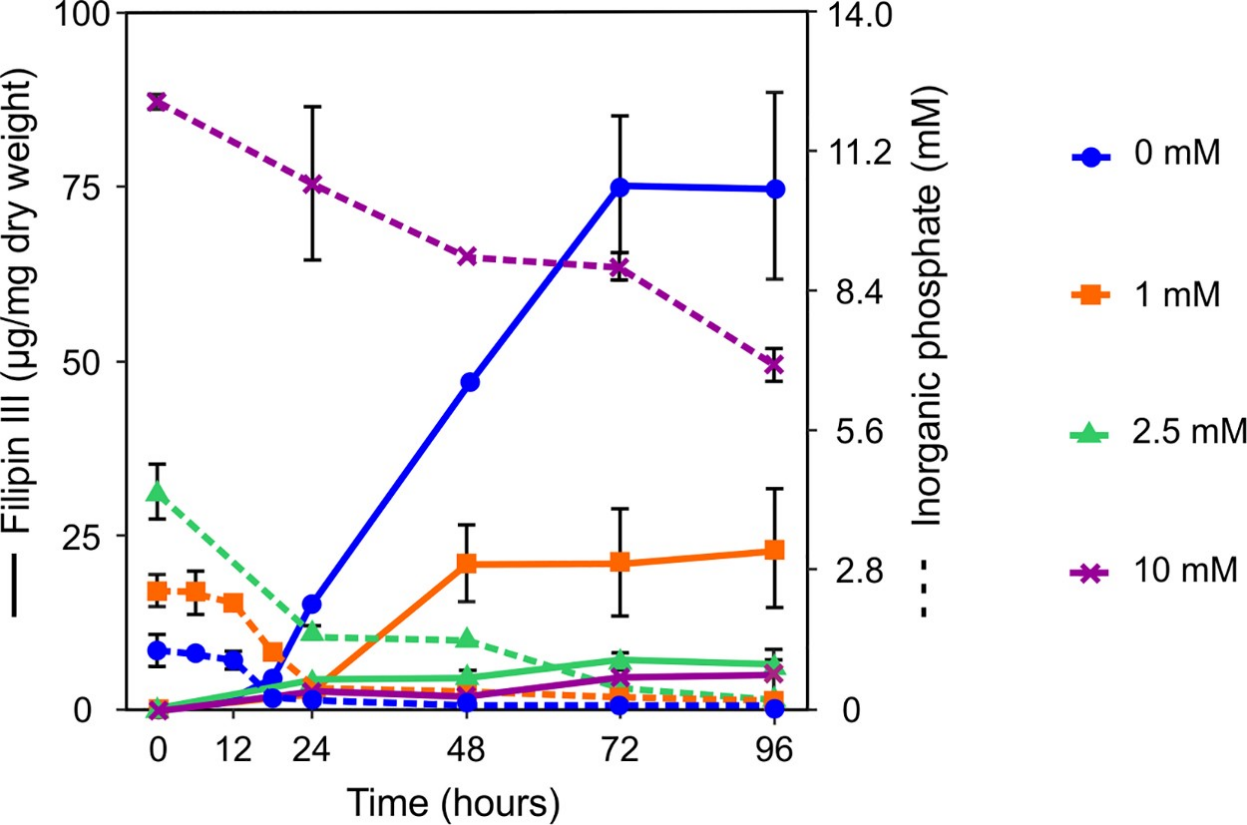
Phosphate consumption in YEME medium by *S*. *filipinensis*. Fermentations were carried out at 30°C in YEME medium supplemented with variable concentrations of inorganic phosphate (0, 1, 2.5 and 10 mM). The consumption of inorganic phosphate (broken lines) and the specific production of filipin (solid line) were determined. The vertical bars represent the standard deviation between three biological replicates.

Given the paucity of data regarding the effect of inorganic phosphate (Pi) on filipin production we assayed increasing concentrations of Pi (1 to 10 mM) added at inoculation time. The addition of growing concentrations of Pi reduced drastically the specific production (filipin per unit of dry weight) of this macrolide in yeast extract-malt extract (YEME) medium and at the same time stimulated the growth rate of the culture (Fig 1A). The strong effect of phosphate was in agreement with previous information for other phosphate-controlled polyene macrolides such as pimaricin [33]. Similar results were obtained when exogenous Pi was added at inoculation time to cultures in defined Lechevalier or AGS media (not shown), although the filipin levels obtained in these media were less than 6% of those obtained in complex YEME medium. Because of the drastic effect exerted by 5 mM Pi we studied the effect of adding lower concentrations of phosphate to the medium. This approach led us to determine that the addition of 2.5 mM Pi was enough to reduce filipin production by 90% at 72 hours of growth. Fig 1B shows that higher Pi concentrations did not cause a greater decrease in production, indicating the saturation of phosphate control. Measurement of Pi in fresh non-supplemented YEME medium rendered values around 1.3 mM, and during growth *S*. *filipinensis* consumed this phosphate at 18 hours of fermentation, descending to values of 0.01 mM (Fig 2).

These results demonstrate that filipin production is highly sensitive to phosphate control, in contrast to the lower sensitivity of other classes of secondary metabolites such as orthosomycin antibiotics [38] or cephalosporins [39] that are only inhibited at high phosphate concentrations (20–100 mM). In this respect, the high sensitivity of filipin biosynthesis to phosphate is highly similar to that of other polyketides including tetracyclines [40], or macrolides like spiramycin [41] and pimaricin [33].

### Transcription of *fil* genes is repressed by phosphate

**Fig 3:**
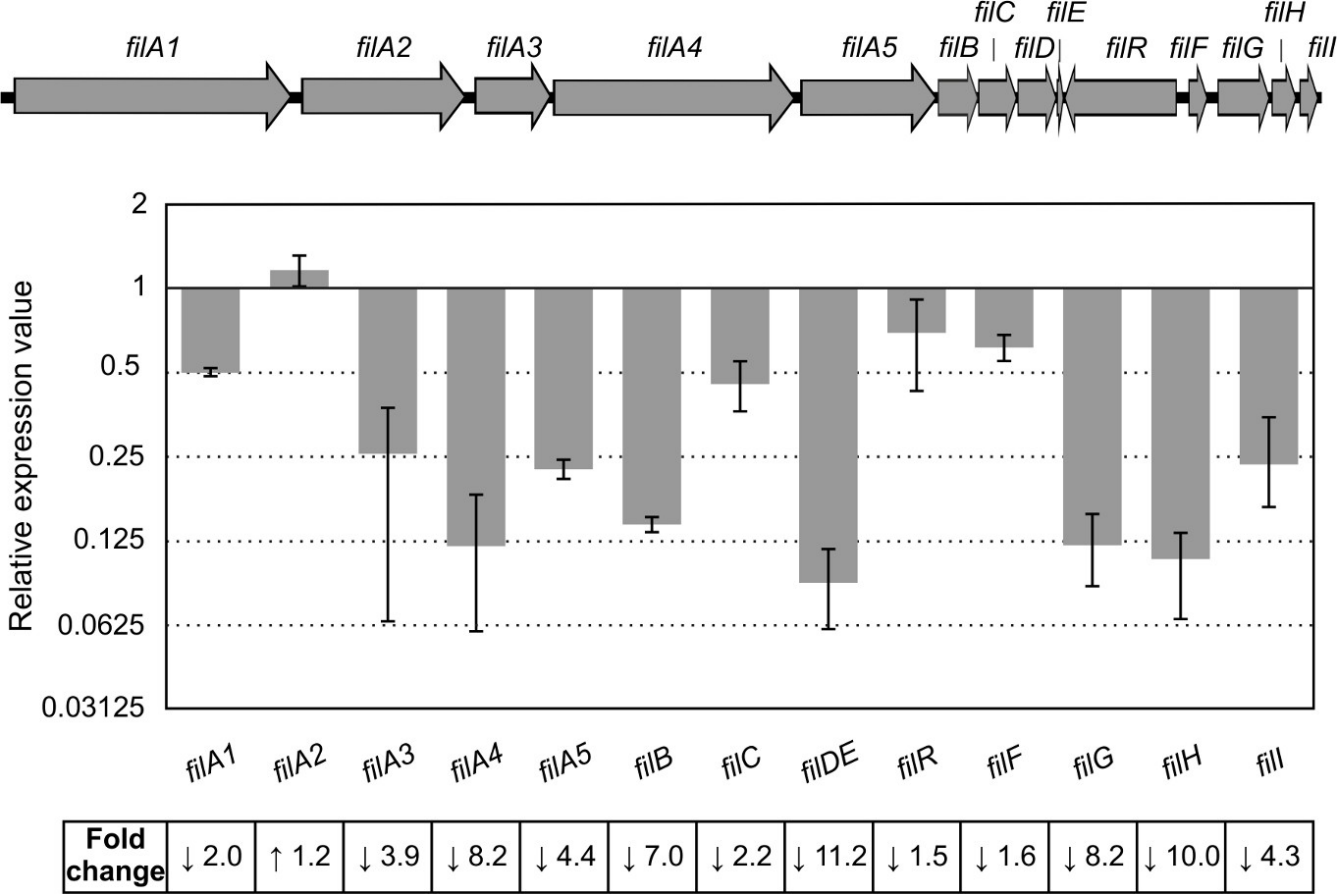
Gene expression analysis of *fil* genes in the presence of 10 mM added phosphate. Gene expression was assessed using RT-qPCR with the primers indicated in S1 Table. Transcriptional analysis of *filD* and *filE* was performed jointly because both genes are transcribed together and *filE* is small. The relative values are referred to 1, the assigned relative value for the expression of each gene in the absence of added phosphate. The expression of *rrnA1* (encoding 16S rRNA) was used as control. Error bars were calculated by measuring the standard deviation of the ratio value among three biological and three technical replicates of each sample. The RNA templates were from 48 h cultures grown in YEME medium without sucrose. Fold change values are indicated below. Chromosomal arrangement of *fil* genes is indicated on the top.

The degree of expression of the filipin biosynthetic genes in control (unsupplemented) and phosphate-supplemented cultures was studied by reverse transcription-quantitative polymerase chain reaction (RT-qPCR). Total RNA was prepared from *S*. *filipinensis* after growth for 48 h in YEME medium (when filipin is actively produced) in the absence or presence of added phosphate (10 mM), and used as template for gene expression analysis. The expression levels of all *fil* genes in the presence of phosphate in relation to those in its absence (assigned a relative value of 1) are shown in Fig 3. Primers were specific to sequences within the 14 *fil* genes [18] and were designed near the 5´-end of the gene (S1 Table).

Phosphate addition to the culture medium resulted in a general decrease in the expression of almost all the genes of the filipin gene cluster, suggesting that the promoters that control the expression of those genes are subject to phosphate modulation. Only *filA2*, coding for one of the polyketide synthases, did not show a decreased expression under conditions of excess phosphate. The distinct transcription of *filA2* when compared with other *fil* genes can be explained because this gene is transcribed as a monocistronic transcript (unpublished), although the reason why it is not repressed by high phosphate remains unknown. The rest of the structural biosynthetic genes (*filA1*, *filA3*, *filA4* and *filA5*) were clearly underexpressed in the presence of 10 mM Pi. Similarly, the transcription of remaining genes of the cluster was also affected negatively, including the three regulatory genes *filR*, *filF* and *filI* (Fig 3). These results demonstrate that the negative control of filipin biosynthesis by phosphate is exerted, at least, at the transcription level, as occurs with other polyenes like pimaricin or candicidin [33,42].

### Cloning of the *phoR-phoP* cluster

**Fig 4:**
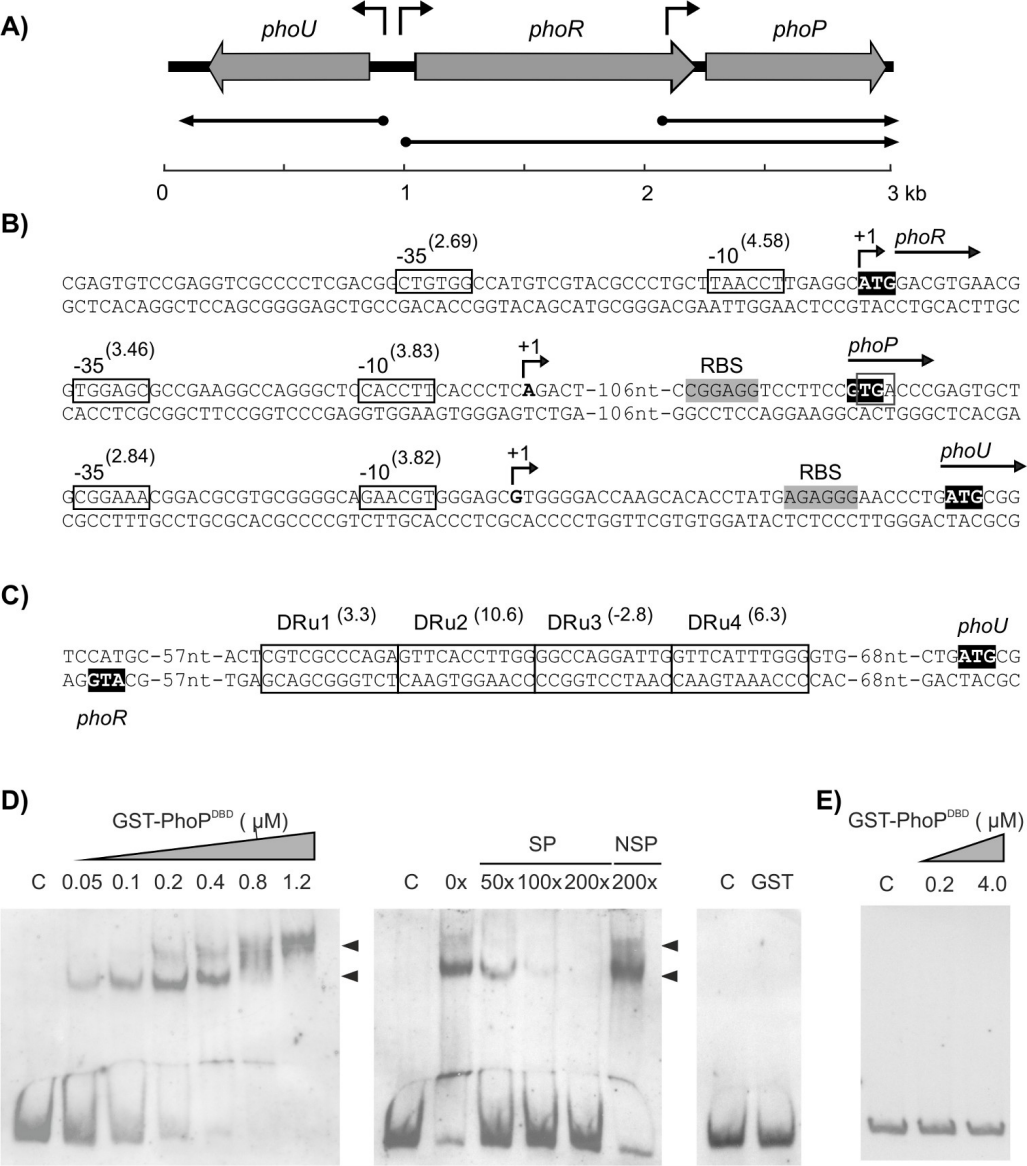
DNA region containing the *phoR*-*phoP* cluster of *S*. *filipinensis*. A) Pointed boxes indicate the size and orientation of the *phoU*, *phoR*, and *phoP* genes. Arrows indicate deduced transcriptional units. B) Transcriptional start point of promoters. The position of the transcriptional start point was determined by 5′ RACE. The putative -10 and -35 hexanucleotides are boxed. Scores resulting from the comparison to the matrices reported by Bourn and Babb [43] for *Streptomyces* are indicated between brackets. The TSP is indicated by a bent arrow and bold type letter. Nucleotides showing similarity with the 16S RNA, which could form a ribosome-binding site, are shaded and labelled RBS. C) PhoP binding sequences in the *phoU*-*phoRP* intergenic region. The localized direct repeats (DRus) are indicated by boxes using the model II scoring matrix described by Sola-Landa et al. [44]. The information content (Ri) in bits [45] of each repetition unit is indicated in parentheses. The ATG triplets corresponding to the amino terminal end of PhoU and PhoR are highlighted in black. D) Binding of pure GST-PhoP^DBD^ to the intergenic *phoU*-*phoR* region as shown by EMSAs. The DNA fragment was labeled with digoxigenin and mixed with increasing concentrations of GST-PhoP^DBD^ (0 to 1.2 μM) (left panel). The middle panel shows a competition experiment between labelled (0.08 ng/μl) and growing concentrations of unlabelled probe using 0.4 μM GST-PhoP^DBD^. The right panel shows a control with pure GST protein. C: control without protein; NSP: non-specific probe; SP: specific probe. DNA-protein bands are indicated by arrows. E) EMSA assay with the *phoP* second promoter region. Conditions were as in D.

The *phoR-phoP* cluster of *S*. *filipinensis* was identified by screening the *S*. *filipinensis* DSM 40112 cosmid library [18] with a 1971 bp BamHI-PstI probe encompassing the homologous *phoP* and the 3´-end of *phoR* genes from *S*. *lividans* [22]. Several cosmids were found to hybridize with the probe, and one of them (12F5) was chosen for sequencing. A 2979 bp nucleotide sequence was found to comprise the *phoU-phoRP* cluster, with an organization identical to that found in *S*. *lividans*, *S*. *coelicolor*, *S*. *avermitilis*, or *S*. *natalensis* [22,24,33,35]. Thus, the genes encoding the two-component system *phoR* and *phoP*, and the modulator of phosphate response *phoU* were located in opposite orientation (Fig 4A). The *phoR* gene in *S*. *filipinensis* encodes a 418 amino-acid sensor kinase, whereas the *phoP* gene encodes a 223 amino-acid response regulator. Both proteins were very similar to their counterparts from other *Streptomyces* spp., thus PhoP showed ca. 98% identity to the PhoP proteins from *S*. *lividans*, *S*. *coelicolor*, *S*. *avermitilis*, or *S*. *natalensis*, while PhoR had a slightly lower identity, ranging from 88% to *S*. *natalensis* PhoR to 93% to *S*. *avermitilis* PhoR (S1 and S2 Figs).

### Two promoters control *phoP* transcription

Because of their chromosomal arrangement, *S*. *filipinensis phoU* and *phoR* genes must be transcribed from divergent promoters present in their intergenic region, while *phoP*, could be expressed either as a bicistronic transcript from the *phoR* promoter or as a monocistronic transcript from a dedicated promoter internal to the coding sequence of *phoR* as it has been described in *S*. *lividans* [31]. To determine the transcriptional start points of these putative promoters, 5′-RACE experiments were carried out. Once the +1 sites were known, the corresponding -10 and -35 boxes of each promoter were established by comparison with the matrices reported by Bourn and Babb [43] for *Streptomyces* that take into account the nucleotides occurring in 13-nucleotide stretches, including the -10 or -35 consensus hexamers (see Materials and methods). Results are summarized in Fig 4B.

The *phoR* transcription start point (TSP) is located at the adenine of the ATG codon (S3 Fig), thus indicating that this gene is transcribed as a leaderless mRNA and that the starts of transcription and translation coincide in *phoR* as occurs in *S*. *coelicolor* and *S*. *lividans* [24,31]. Analysis of the upstream sequence revealed TAACCT and CTGTGG as the -10 and -35 boxes (scores 4.58 and 2.69, respectively). Both boxes are separated by 19 nucleotides, with the -10 hexamer centred at 9 nucleotides from the TSP (Fig 4B). For its part, the *phoP* TSP located at the 3´-region of *phoR* was identified at an adenine 124 nucleotides upstream from the GTG start codon (S3 Fig). The analysis of the upstream sequence using the matrices of Bourn and Babb revealed a clear promoter, with the -10 box CACCTT (score 3.83) located 10 nucleotides upstream from the observed TSP and the -35 box TGGAGC (score 3.46) separated by 17 nucleotides. In this case, a putative ribosome binding site was identified 10 nucleotides upstream from the GTG start codon (Fig 4B). In the case of *phoU*, the TSP was located at a guanine situated 35 nucleotides upstream from the ATG codon (S3 Fig). The -10 and -35 boxes (GAACGT and CGGAAA respectively) were centred at positions -9 and -32 from the TSP and are separated by 17 nucleotides. A putative ribosome binding site was identified 10 nucleotides upstream from the ATG start codon (Fig 4B).

The possible co-transcription of *phoR* and *phoP* was studied by reverse transcriptase-polymerase chain reaction experiments. Total RNA was prepared from *S*. *filipinensis* after growth for 48 h in YEME medium. Primers were designed to obtain cDNA corresponding to unabated transcription between both genes (see Materials and methods), the forward primer hybridizing at the 3´-region of *phoR* upstream the leader sequence of *phoP* and the reverse primer at 5´-end region of *phoP*. Results showed an amplification band of 351 bp as expected, indicating that *phoP* was co-transcribed with *phoR*. Consequently, two different promoters control *phoP* transcription, one directing the transcription of the *phoRP* operon, and the second one dedicated to the individual transcription of *phoP*.

### Two PHO boxes are present in the *phoRP-phoU* bidirectional promoter region

In other *Streptomyces* species, PhoP directly controls the expression of the *phoRP* system by binding to the *phoRP*-*phoU* intergenic region at specific nucleotide sequences called PHO boxes [24,33,35]. Bioinformatic analysis of the *phoRP*-*phoU* intergenic region in *S*. *filipinensis* revealed the presence of two PHO boxes, each consisting of two repeated sequences of 11 nucleotides (DRu1-DRu4) in the *phoU* direction (Fig 4C). Conservation of DRus was studied by using the model II scoring matrix described by Sola-Landa et al. [44], and the information content (*R*i) of each DRu was calculated using the theory-based model described by Schneider [45]. On this basis, DRu1-DRu4 presented *R*i values of 3.3, 10.6, -2.8 and 6.3 bits respectively (Fig 4C). As a consequence, they would conform to the CCE_U_E_S_ model of PhoP binding to its target sequences [25]. According to this model, PhoP would bind with high affinity to DRu1 and DRu2 (C, core sequences) and this interaction would allow the subsequent association of other PhoP molecules to the next, less conserved PHO box, through stabilization by protein-protein interactions [25]. Noteworthy, the DRu4 is only 4 nucleotides away from the -35 hexamer of *phoU* promoter. No PHO boxes were found in the *phoR* coding region thus suggesting that PhoP does not control the transcription from *phoP* second promoter.

The presence of two PHO boxes in the *S*. *filipinensis phoRP* promoter is consistent with the number of boxes found in *S*. *coelicolor* [24] or *S*. *avermitilis* [35], whereas in *S*. *natalensis* four boxes have been reported [33]. The presence of four boxes in the *S*. *natalensis* promoter led to suggest that it could be the cause of the strong repression by phosphate detected in the production of the antifungal pimaricin [33], but at least other factors must also be involved, since the production of filipin by *S*. *filipinensis* also undergoes a similar drastic reduction due to the presence of phosphate in the culture medium. Therefore, the number of PHO boxes does not seem to influence the degree of repression by phosphate.

### PhoP binds *phoRP* promoter, but not *phoP* promoter

In order to confirm the binding of PhoP to the *phoU*-*phoR* intergenic region of *S*. *filipinensis* we performed electrophoretic mobility shift assays (EMSAs) with GST-PhoP^DBD^ from *S*. *coelicolor* [24], which shares a 97.4% identity with the DNA-binding domain of *S*. *filipinensis* PhoP, and a labelled 298 bp DNA fragment containing this region (see Materials and methods). This protein has an affinity for DNA similar to the complete protein and binds to the PHO boxes in a constitutive manner [24]. As shown in Fig 4D, GST-PhoP^DBD^ bound to this region resulting in the formation of two DNA-protein retardation bands, in agreement with the existence of two PHO boxes in the intergenic region. The intensity of the retarded band(s) was diminished by the addition of the same unlabelled DNA, but not by non-specific competitor DNA, and increased when a higher concentration of protein was used, indicating that the interaction was specific (Fig 4D). Control reactions made with pure GST protein were negative, excluding a possible binding of this protein to the promoter (Fig 4D).

To study the putative binding of GST-PhoP^DBD^ to *phoP* dedicated promoter, a similar experiment was carried out using a 351 bp labelled probe covering that region (see Materials and methods). The results confirmed the absence of PhoP binding to this promoter (Fig 4E), indicating that expression form this promoter is independent of PhoP as occurs in *S*. *coelicolor* [24].

### Inactivation of *phoP* or *phoRP* increases filipin production and reduces sensitivity to phosphate repression

**Fig 5:**
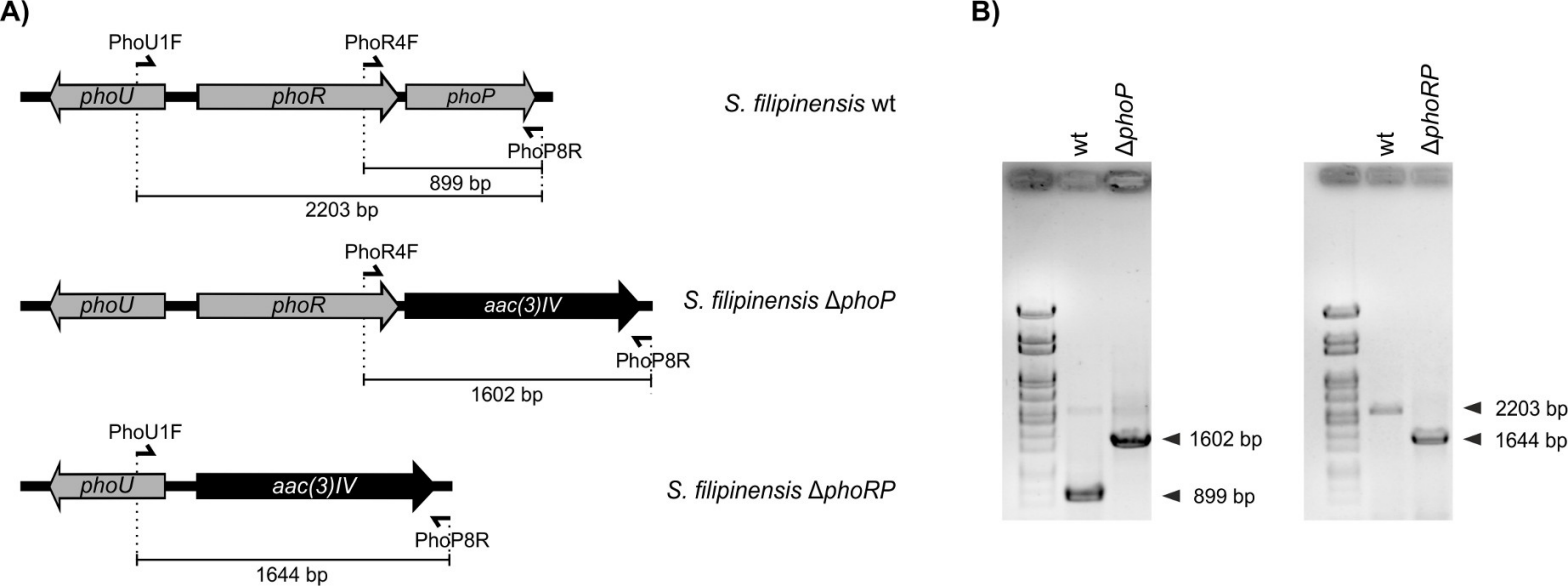
Construction of *phoP* and *phoRP* mutants. A) Predicted PCR fragment amplification of the parental strain and the different mutants. The primers used in the assay are indicated with arrowheads. The *acc(3)IV-oriT* cassette is indicated in black. B) PCR analysis of the wild type and the mutants.

To determine the function of *phoP*, we deleted it by using the REDIRECT gene replacement technology as indicated in “Materials and methods”. Double-crossover mutants were screened by apramycin resistance, and further verified by PCR analysis (Fig 5). The same strategy was used for the deletion of both *phoR* and *phoP* (Fig 5).

**Fig 6:**
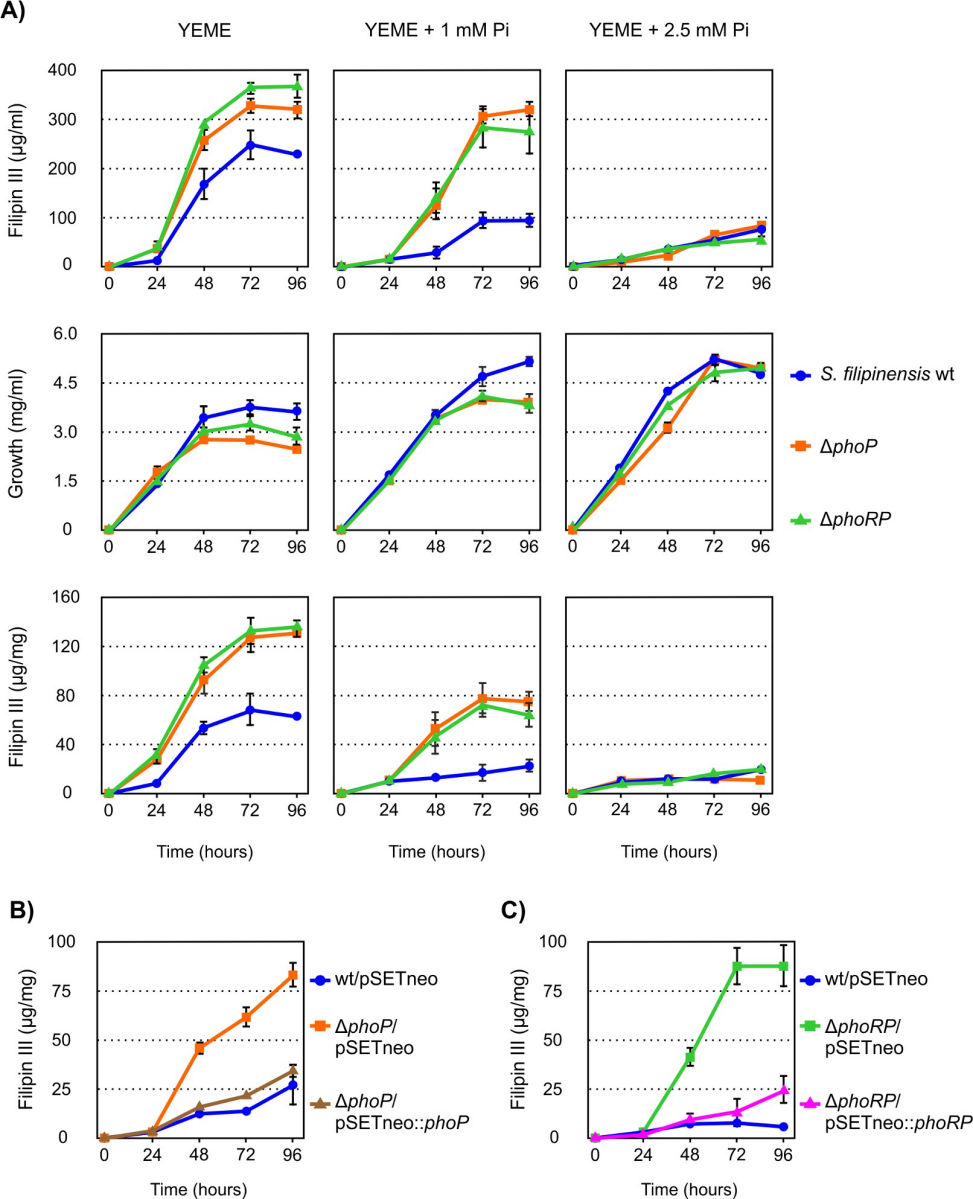
Both PhoP or PhoRP inactivation increase filipin production and gene complementation restores it. A) Time course quantification of filipin III production and growth curves in the wild-type and mutant strains. Fermentations were carried out at 30°C in YEME medium supplemented with variable concentrations of inorganic phosphate (0, 1 and 2.5 mM). B and C) Effects of gene complementation in YEME medium in the presence of 1 mM supplemented phosphate. Data are the average of three duplicate flasks. Vertical bars indicate standard deviation of the mean values.

In order to study the effect that the inactivation of the *phoP* and *phoRP* genes had on the production of filipin, fermentation broths produced by the new mutant strains, when grown in YEME medium supplemented with different concentrations of Pi (0, 1 and 2.5 mM), were extracted with methanol and analysed for the presence of filipin III (the major component of the filipin complex). Results showed that both mutants behaved as filipin overproducers in the absence of added phosphate, reaching a 130 to 145% of the filipin produced by the wild type strain (Fig 6A). Moreover, when the medium was supplemented with 1 mM Pi, polyene biosynthesis by the deleted strains maintained approximately the same levels at late culture times (~300 μg/ml) while the parental strain underwent the effects of phosphate inhibition and production was reduced to half of that obtained under the non-supplemented conditions. Only when we added 2.5 mM Pi, filipin production showed no significant differences between the three strains, suggesting that at this phosphate concentration the mutant strains are still sensitive to phosphate repression. This suggests that there must exist alternative mechanisms to phosphate control other than PhoRP. Antibiotic production in *S*. *natalensis*, *S*. *lividans* and *S*. *lydicus* Δ*phoP* mutants is also sensitive to high Pi concentrations [22,33,34], thus it seems likely that a yet unidentified PhoP-independent mechanism blocks antibiotic biosynthesis under excess phosphate conditions.

Overproduction of antibiotics upon *phoP* deletion under phosphate limitation has also been reported in four species. Thus, *S*. *lividans* overproduces actinorhodin and undecylprodigiosin [22], *S*. *natalensis* and *S*. *lydicus* overproduce pimaricin [33,34], and *S*. *avermitilis* overproduces avermectin [35]. Surprisingly, in the model streptomycete *S*. *coelicolor*, *phoP* deletion accelerates actinorhodin production on complex media without overproducing it [36], whilst reduces actinorhodin and undecylprodigiosin production when using defined media [37].

### Gene complementation restores filipin production to wild type levels

To confirm that the gene deletions were directly responsible for the overproduction of filipin III and the reduced sensitivity to phosphate repression, we complemented all mutants with the corresponding gene/s. For that purpose, we introduced one copy of *phoP* into the genome of *S*. *filipinensis* Δ*phoP* using the integrative plasmid pSETneo::phoP (see materials and methods). pSETneo was also introduced into *S*. *filipinensis* as control. Interestingly, introduction of the vector diminished the ability of *S*. *filipinensis* Δ*phoP* to produce filipin III, almost restoring it to parental strain levels, particularly in the presence of 1 mM added Pi (Fig 6B). Similar results were obtained when we introduced one copy of *phoRP* into the genome of *S*. *filipinensis* Δ*phoRP* using the integrative plasmid pSETneo::phoRP (see Materials and methods) (Fig 6C). Restoration of antifungal production close to wild type levels indicates that control of filipin production by phosphate is mediated by PhoRP in *S*. *filipinensis*.

### PhoP represses *fil* genes expression indirectly

In order to study if the increase in filipin production in the mutants was a direct consequence of a higher transcription of filipin biosynthetic genes, we performed expression studies by RT-qPCR. Since both the *phoP* and *phoRP* deletions caused the same phenotype, the strain *S*. *filipinensis* Δ*phoP* was chosen for the analyses. Total RNA was prepared from *S*. *filipinensis* Δ*phoP* after growth for 48 h in YEME medium supplemented with 1 mM Pi, conditions where the highest production differences had been observed, and used for analysis. The transcriptional levels of each gene in the mutant strain were compared with those of the wild strain, to which a relative expression value of 1 was assigned. Primers used are indicated in S1 Table.

**Fig 7:**
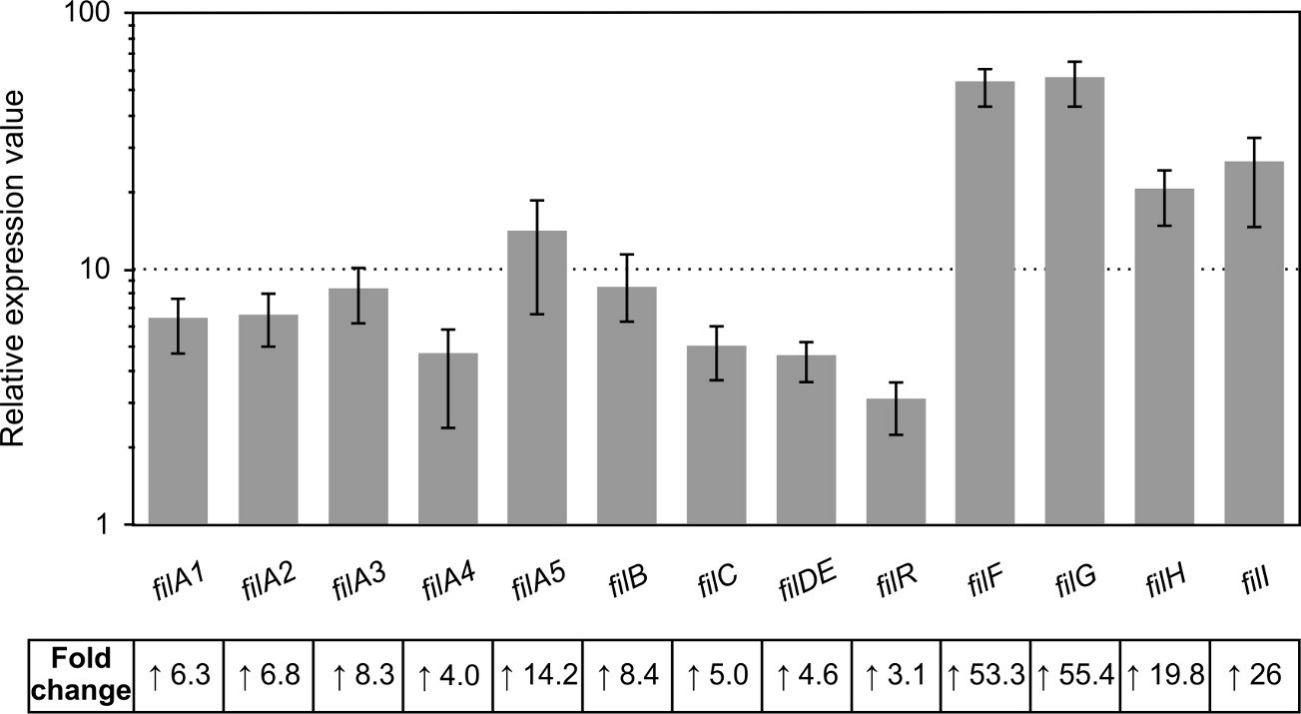
Gene expression analysis of *fil* genes in *S*. *filipinensis* Δ*phoP*. Gene expression was assessed by RT-qPCR with the primers indicated in S1 Table. The RNA templates were from 48 h cultures grown in YEME medium without sucrose supplemented with 1 mM phosphate. Relative values are referred to 1, the assigned relative value for the expression of each gene in the parental strain. The expression of *rrnA1* was used as control. Error bars were calculated by measuring the standard deviation of the ratio value among three biological and three technical replicates of each sample. Fold change values are indicated below.

In agreement with the filipin production observed in the mutant, *phoP* deletion caused a significant increase in the expression of all *fil* genes (Fig 7), which indicates that PhoP is a repressor of filipin production. The polyketide synthase genes *filA1*-*filA5* had transcription values between 4 and 14-fold. The transcription of *filR*, the SARP-LAL positive regulator of the cluster, was the least affected by the *phoP* deletion showing a 3-fold increment in transcription in the mutant strain. On the other side, the genes that presented a more relevant variation were those located upstream from *filR*, with increments in expression between 20- and 55-fold (Fig 7). Repression of polyene biosynthetic genes by PhoP has only been studied previously in *S*. *natalensis*. Contrary to what occurs in *S*. *filipinensis*, in that strain the transcription of only four genes was affected upon *phoP* deletion, and none of them was a cluster situated regulatory gene [33].

**Fig 8:**
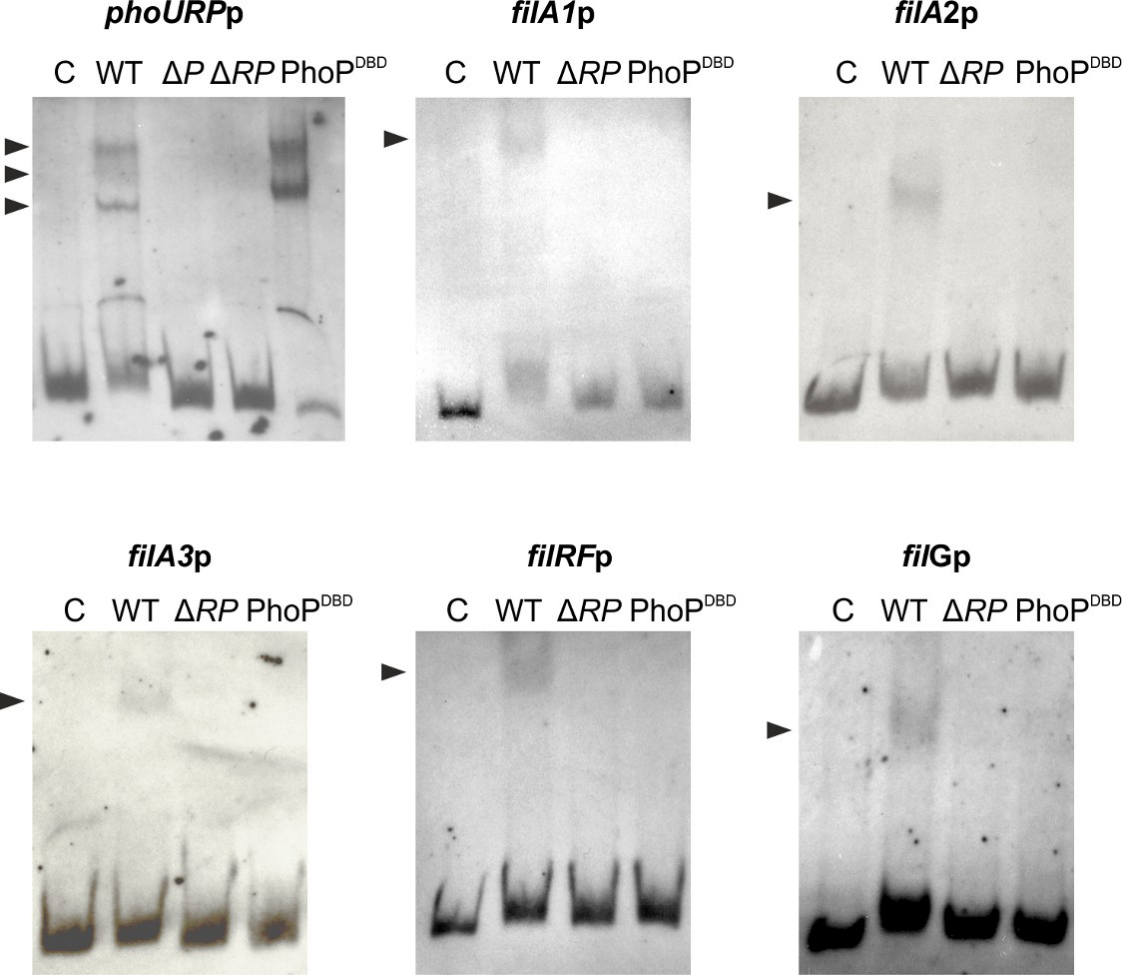
Transcriptional regulation of PhoP of filipin biosynthesis is indirect. EMSAs of protein extract binding to the promoter regions of *filA1*, *filA2*, *filA3*, *filRF*, *filG* and *phoURP*. The arrows indicate the DNA–protein complexes. All experiments were carried out with 0.08 ng/μl labeled DNA probe. The probes were incubated for 30 min at 30°C with 30 μg of protein extract prepared from cultures of *S*. *filipinensis* and *S*. *filipinensis* Δ*phoRP* grown for 48 h on YEME medium. Binding of 0.4 μM pure GST-PhoP^DBD^ was used as control. Promoter regions are indicated at the top of each panel. C: control without protein.

Bioinformatic analysis of the sequence of intergenic regions within the *fil* cluster revealed no PHO boxes, thus suggesting that PhoP cannot bind to these regions, and that the repression exerted by PhoP must be indirect via a second transcriptional regulator, as has been suggested in *S*. *natalensis* [33]. PhoP absence of binding was confirmed by EMSAs with *S*. *coelicolor* GST-PhoP^DBD^ and the promoter regions of selected *fil* genes, whereas two retardation bands were observed when we used the *phoURP* promoter (Fig 8). In order to confirm that the binding of the putative transcriptional regulator controlled by PhoP was PhoP dependent, we performed EMSA experiments using cell-free extracts prepared from the wild-type and the mutant strains grown in YEME medium for 48 hours and the promoter regions of *fil* genes. Interestingly, results showed retardation bands in the parental strain that were absent in the mutants with all the promoter regions assayed, thus suggesting that binding of this unknown regulator or regulators was PhoP dependent (Fig 8). The *phoU*-*phoRP* intergenic region was also tested as control, showing at least two retardation bands in the wild-type strain that could be produced by PhoP. A third retardation band is also observed in the parental strain suggesting that another PhoP dependent transcriptional regulator could be interacting with this region. Fig 8 shows the results observed with the Δ*phoRP* mutant, identical results were observed with the Δ*phoP* mutant (not shown).

### PhoP regulates morphological differentiation

**Fig 9:**
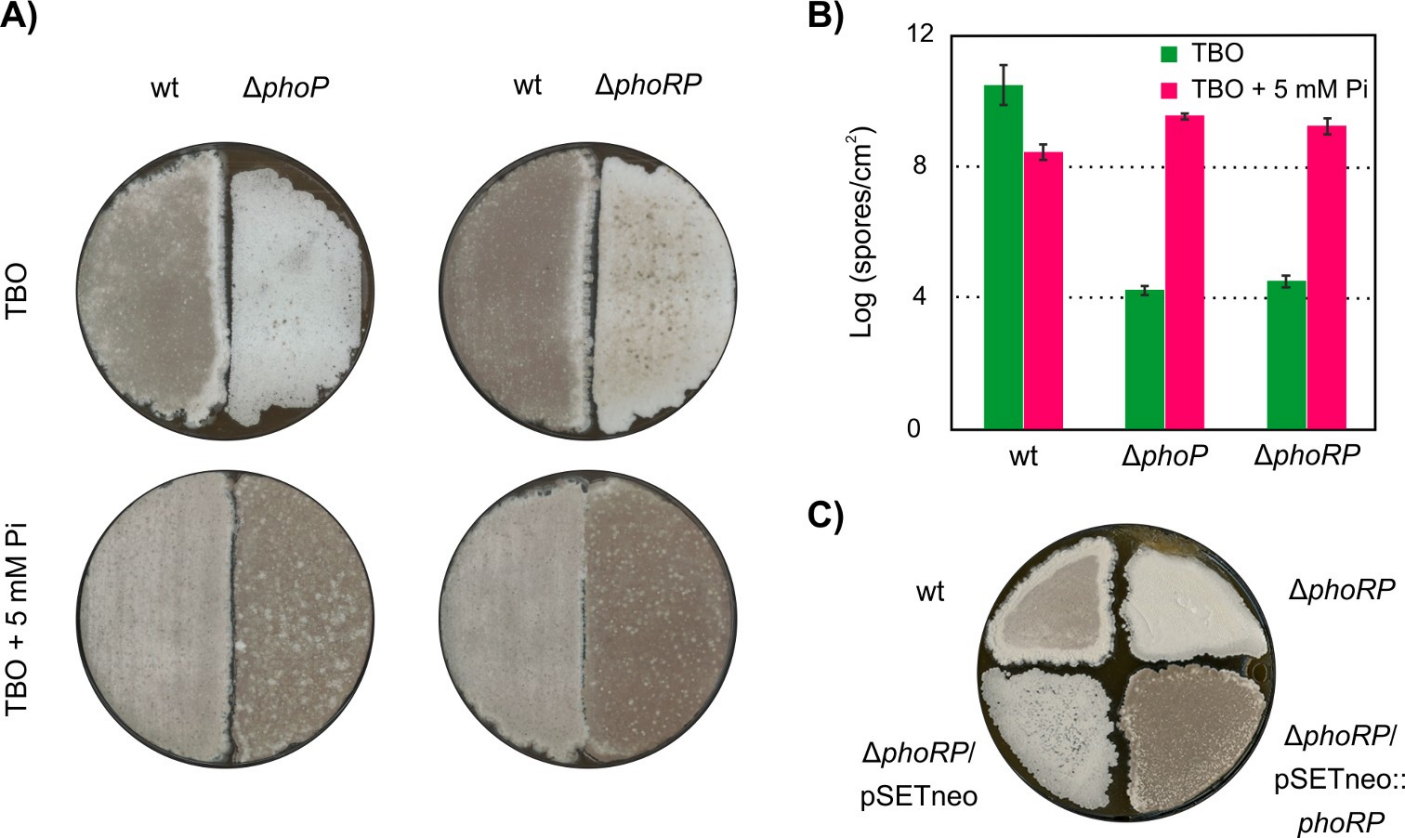
The effect of *phoP* or *phoRP* deletion on sporulation. A) Phenotype of the parental and the mutant strains plated on TBO and TBO supplemented with 5mM Pi agar followed by incubation at 30°C for 9 days. B) Spore counts (per square cm) under the same conditions. Data are the average of five replicates. C) Effect of gene complementation on spore formation.

Inactivation of *phoRP* in *S*. *filipinensis* produced remarkable changes in the patterns of morphological differentiation (Fig 9A). When grown on TBO (tomato paste-baby oatmeal) medium, the wild-type strain initiates aerial mycelium formation around 24 hours of growth and sporulation after 3–4 days of incubation. Both mutants, however, although started differentiation at about the same time, were severely impaired in viable spore formation even after 9 days of incubation, rendering viable spore counts ca. 10^6^ times lower than the parental strain (Fig 9A and Fig 9B). In *S*. *avermitilis*, Δ*phoP* mutants also produce fewer spores under conditions of phosphate shortage, but additionally they grow poorly, and these effects are reversed in media supplemented with Pi [35]. In this case, the reduced expression of genes involved in phosphate scavenging, transport, and storage from organic sources which has been shown to be dependent on PhoP is sufficient to explain such phenotypes [26,27,46]. But in *S*. *filipinensis*, there is not an important difference in growth of the mutant strains when compared to the parental strain (Fig 6), thus suggesting that other genes must be affected.

Interestingly, when the medium was supplemented with 5 mM Pi, the mutants showed a rapid and abundant formation of spores, resulting in spore counts 4–8 times higher than the parental strain under the same conditions, which showed a scarcer sporulation (ca. 100-fold decrease) than in the non-supplemented medium (Fig 9A and Fig 9B). The introduction of a functional copy of *phoRP* in the mutant Δ*phoRP* restored its sporulation ability to that of the wild-type strain (Fig 9C). Similar results were observed when we complemented Δ*phoP* mutant (not shown). These results indicate that there is a clear relationship between the phosphate starvation response mediated by PhoP and the sporulation process in *S*. *filipinensis*.

The slower morphological differentiation (manifested by poor sporulation) of the wild-type strain in phosphate abundance compared to Pi limitation could be attributed to a lower presence of the PhoRP system given that the transcription of both genes is repressed in phosphate abundance (our unpublished results), and was expected given that phosphate shortage is one of the main factors that triggers morphogenesis [47]. But surprisingly, under the same phosphate abundance conditions, the Δ*phoRP* and Δ*phoP* mutants showed a more abundant spore production than the parental strain, approximately 10 fold more and only 10 fold less than the one achieved by the parental strain under Pi-limiting conditions (Fig 9B). This was unexpected, and demonstrates that the availability of a functional PhoP protein is not the only key factor involved in the sporulation process and that other regulatory pathways take the control when the PhoRP system is absent.

## Materials and methods

### Microbial strains and cultivation

*S*. *filipinensis* DSM 40112 was routinely grown in YEME medium [48] without sucrose. Sporulation was achieved as described elsewhere [49]. *Escherichia coli* strain DH5α was used as a host for DNA manipulation. *E*. *coli* BW25113 [pIJ790] was used for gene replacement experiments. *E*. *coli* ET12567 [pUZ8002] was used as donor in intergeneric conjugations. Determinations of inorganic phosphate in culture media were carried out as described by Lanzetta et al. [50]. For spore capacity determination, 2.5x10^6^ fresh spores of the mutants and the parental strain were plated onto the surface of plates containing TBO medium [51], and plates were incubated at 30°C for 9 days. After harvesting the resulting spores, spore counting was done by plating serially diluted spore suspension on TSA (tryptic soy agar) medium [52].

### Genetic procedures

Intergeneric conjugations between *E*. *coli* ET12567[pUZ8002] and *S*. *filipinensis* were performed as described [53]. pUC19 (New England Biolabs) was used as the routine cloning vector, and pSETneo (Am^R^, Neo^R^, pUC18 replicon, ϕC31 *attP* [54]) was used for intergeneric conjugations. Plasmid DNA preparation, DNA digestion, fragment isolation, and transformation of *E*. *coli* were performed by standard procedures. Polymerase chain reactions were carried out using Hybrid DNA polymerase as described by the enzyme supplier (EURx). DNA sequencing was accomplished by the dideoxynucleotide chain-termination method using the DYEnamic ET Terminator Cycle Sequencing Kit (GE Healthcare) with an Applied Biosystems ABI 3130XL DNA genetic analyzer (Foster City, CA., USA).

### Isolation of total RNA and reverse transcription-PCR

RNA was extracted as described in detail elsewhere [16]. Transcription was studied as described [55]. Briefly, we used the SuperScript One-Step reverse transcriptase-PCR (RT-PCR) system with Platinum Taq DNA polymerase (Invitrogen) and 150 ng of total RNA as template. Conditions were as follows: first strand complementary DNA (cDNA) synthesis, 50°C for 40 min followed by heating at 94°C for 2 min; amplification, 35 cycles of 94°C for 40 s, 63°C for 30 s, and 72°C for 30 s. Primers PhoR4F 5’-GCACGGCGGGGAGGTCAC-3’ and PhoP6R 5’-CGGCAGCATCAGGTCGAGGAG-3’ were designed to detect the possible co-transcription of *phoR* and *phoP*. Negative controls were carried out with each set of primers and Platinum Taq DNA polymerase in order to confirm the absence of contaminating DNA in the RNA preparations. The identity of each amplified product was corroborated by direct sequencing of the PCR product.

### Reverse transcription-quantitative PCR

Reverse transcription of total RNA was performed as described in detail elsewhere [16] on RNA samples with RNA integrity number (RIN) values [56], ranging from 8.0–9.0. Reactions were carried out on three biological replicates with three technical replicates each and appropriate controls were included to verify the absence of gDNA contamination in RNA and primer-dimer formation. Primers (see S1 Table) were designed to generate PCR products between 80 and 141 bp, near the 5´ end of mRNA. To check the specificity of real-time PCR reactions, DNA melting curve analyses were performed as described [16]. Baseline and threshold values were determined by the StepOnePlus software. C_t_ values were normalized with respect to *rrnA1* mRNA (encoding 16S rRNA). Relative changes in gene expression were quantified using the Pfaffl method [57] and the REST software [58]. The corresponding real-time PCR efficiency (E) of one cycle in the exponential phase was calculated according to the equation E = 10^[-1/slope]^ [59] using 5-fold dilutions of genomic DNA ranging from 0.013 to 40 ng (n = 5 or 6 with three replicates for each dilution) with a coefficient of determination *R*^2^ **>** 0.99 (S4 Fig).

### Rapid amplification of cDNA ends

Transcription start points were identified by using a 5′-RACE system for rapid amplification of complementary DNA (cDNA) ends kit (Invitrogen) following the manufacturer’s instructions (version 2.0) and as described [16], using 5 μg of total RNA for first-strand cDNA synthesis and the gene-specific primers listed in S2 Table.

### Assessment of filipin production

To assay filipin in culture broths, 1 ml of culture was extracted with 1 ml of methanol, and further diluted with methanol to bring the absorbance at 338 nm in the range of 0.1 to 0.4 units. Control solutions of pure filipin III (Sigma) were used as control. To confirm the identity of filipin, an UV-visible absorption spectrum (absorption peaks at 356, 338, 320 and 311 nm) was routinely determined in a Hitachi U-2000 spectrophotometer. Quantitative determination of filipin was performed as previously described [18], using a Mediterranea Sea C18 column (4.6x150 mm, particle size, 3 mm) (Teknokroma).

### Construction of *phoP* and *phoRP* mutants

Deletion of *phoP* from *S*. *filipinensis* chromosome was made by replacing the gene with a cassette containing an apramycin selective marker using a PCR based system [60]. Plasmid pIJ773 containing the apramycin resistance gene and the *oriT* was used as a template. The mutant was constructed using the oligonucleotides PhoP-Red-F 5´-*accgcttcccgccccggaggtcctttccgtgacccgagtg*ATTCCGGGGATCCGTCGACC-3´ and PhoP-Red-R 5´-cccttcggcacggacgggtgtgtgcggtcgtacggccta TGTAGGCTGGAGCTGCTTC-3´ as primers. These two long PCR primers were designed to produce a deletion of *phoP* just after its start codon leaving only its stop codon behind. The 3´ sequence of each primer matches the right or left end of the disruption cassette (shown in uppercase). The extended resistance cassette was amplified by PCR and *E*. *coli* BW25113 [pIJ790] bearing cosmid 12F5 [18] was electro-transformed with this cassette. The isolated mutant cosmid was introduced into non-methylating *E*. *coli* ET12567 containing the RP4 derivative pUZ8002. The mutant cosmid was then transferred to *S*. *filipinensis* by intergeneric conjugation. Double cross-over exconjugants were screened for their apramycin resistance followed by confirmation by PCR. The same strategy was used for the deletion of *phoRP*, but using primers PhoR-Red-F 5´-*tgtggccatgtcgtacgaaatgcttaaccttgaggcatg*ATTCCGGGGATCCGTCGACC-3´ and PhoP-Red-R.

### Construction of plasmids for gene complementation

In order to complement *phoP* replacement mutant, a 899 bp DNA fragment containing the entire *phoP* gene plus its upstream region (containing its discrete promoter) was amplified by PCR with primers PhoR4F (see above) and PhoP8R 5´-CCTTCGGCACGGACGGG-3´ using *S*. *filipinensis* chromosomal DNA as template. The PCR product was cloned into an EcoRV-cut pSETneo [54], and the construction with the gene in the same orientation of the *neo* gene was selected to yield pSETneo::phoP. This would permit gene expression driven from the promoter of the *neo* gene in case the cloned DNA fragment lacked promoter activity.

Similarly, for *S*. *filipinensis* Δ*phoRP* gene complementation, a 2203 bp DNA fragment containing *phoRP* genes plus their promoter region was amplified by PCR with primers PhoU1F 5´-CTCCACCAGACCGTCGCCG-3´ and PhoP8REcoRI 5´- GGAATTCCCTTCGGCACGGACGGG-3. The PCR product was cloned into an EcoRI/EcoRV-cut pSETneo to yield pSETneo:phoRP.

### PhoP-binding and electrophoretic mobility shift assays

Interaction between DNA-binding domain of the response regulator PhoP (PhoP^DBD^) and the PhoP-binding sequences (PHO boxes) was evidenced by the electrophoretic mobility shift assays (EMSAs) performed as reported previously using pure GST-PhoP^DBD^ [24]. The DNA fragments used for EMSAs were amplified by PCR using the primers PhoU1F and PhoR6R for the *phoRP* promoter or PhoR4F and PhoP6R for *phoP* discrete promoter (S3 Table), and *S*. *filipinensis* chromosomal DNA as template.

Crude protein extracts were obtained as described [61] and DNA binding tests were carried out by EMSAs using *fil* genes promoters PCR amplified using primers indicated in S3 Table. All amplification products were sequenced to confirm the absence of any mutations, and then labelled at both ends with DIG oligonucleotide 3´-end labelling kit 2nd generation (Roche Applied Science).

### Bioinformatic analysis

The matrices used to search for regions -35 and -10 were those derived from the alignments of class C and class A promoters of Bourn and Babb [43]. To search for a combination of ‘class C–*n* nucleotides of separation–class A’, we included *n* columns of null values in the combined matrix. To calculate the information content (*R*i value) of individual PHO boxes we used the theory-based model described by Schneider [45].

### Accession numbers

The sequence of the *phoU*-*phoRP* cluster has been deposited in the GenBank database under the accession number MH347448. The sequences used for qPCR are under the accession number MH638267-MH638271 (filipin polyketide synthase cluster region) and KP769541 (filipin tailoring gene cluster region).

## Supporting information

S1 Fig Domain structure and alignment of *S*. *filipinensis* PhoR with other PhoR proteins. A) Predicted PhoR domains. B) Alignment of *S*. *filipinensis* PhoR with its orthologues from *S*. *avermitilis* (BAC71685), *S*. *coelicolor* (CAB77323), *S*. *natalensis* (CAJ45043) and *E*. *coli* (P08400). Identical amino acids in at least three of the five sequences are shaded. The amino acid residues that form boxes H, N, G1, F and G2 are framed by a dashed line and the amino acids that make up the conserved motifs are indicated by an asterisk. Histidine that is autophosphorylated (H165 in *S*. *filipinensis*) is boxed.
(TIF)

S2 Fig Domain structure and alignment of *S*. *filipinensis* PhoP with other PhoP proteins. A) Predicted PhoP domains. B) Alignment of PhoP with its orthologues from *S*. *avermitilis* (BAC71684), *S*. *coelicolor* (CAB77324), *S*. *natalensis* (CAJ45043) and *E*. *coli* (P0AFJ5). Identical amino acids in at least three of the five sequences are shaded. The amino acid residues that make up the phosphorylation domain and the DNA binding domain are framed by a dashed line and the amino acids important for phosphorylation are indicated by an asterisk. The aspartic residue phosphorylated by PhoR (D52) is boxed.
(TIF)

S3 Fig 5´-RACE experiments. Genomic sequences are indicated at the top, and RACE results at the bottom.
(TIF)

S4 Fig Primer efficiency. The efficiency of each set of primers was calculated according to the equation E = 10^[-1/slope]^-1. Using 5-fold dilutions of genomic DNA, the resulting Ct values were plotted against the logarithm of the DNA as shown in A) for *filH*, *filA2*, *filR* and *rrnA1*. Data are from three replicates, values represent the mean and the vertical bars ± SD. Panel B summarizes information obtained from all plotted data.
(TIF)

S1 Table Primers used for reverse transcription-quantitative PCR. (DOCX)

S2 Table Primers used for rapid amplification of cDNA ends. (DOCX)

S3 Table Primers used for probe amplification for EMSAs. (DOCX)
