# Supplementary figures and images for: Phosphate effect on filipin production and morphological differentiation in Streptomyces filipinensis and the role of the PhoP transcription factor
Source: PLoS One. 2018 Dec 6;13(12):e0208278. doi: 10.1371/journal.pone.0208278 (PMC6283541; doi:10.1371/journal.pone.0208278)

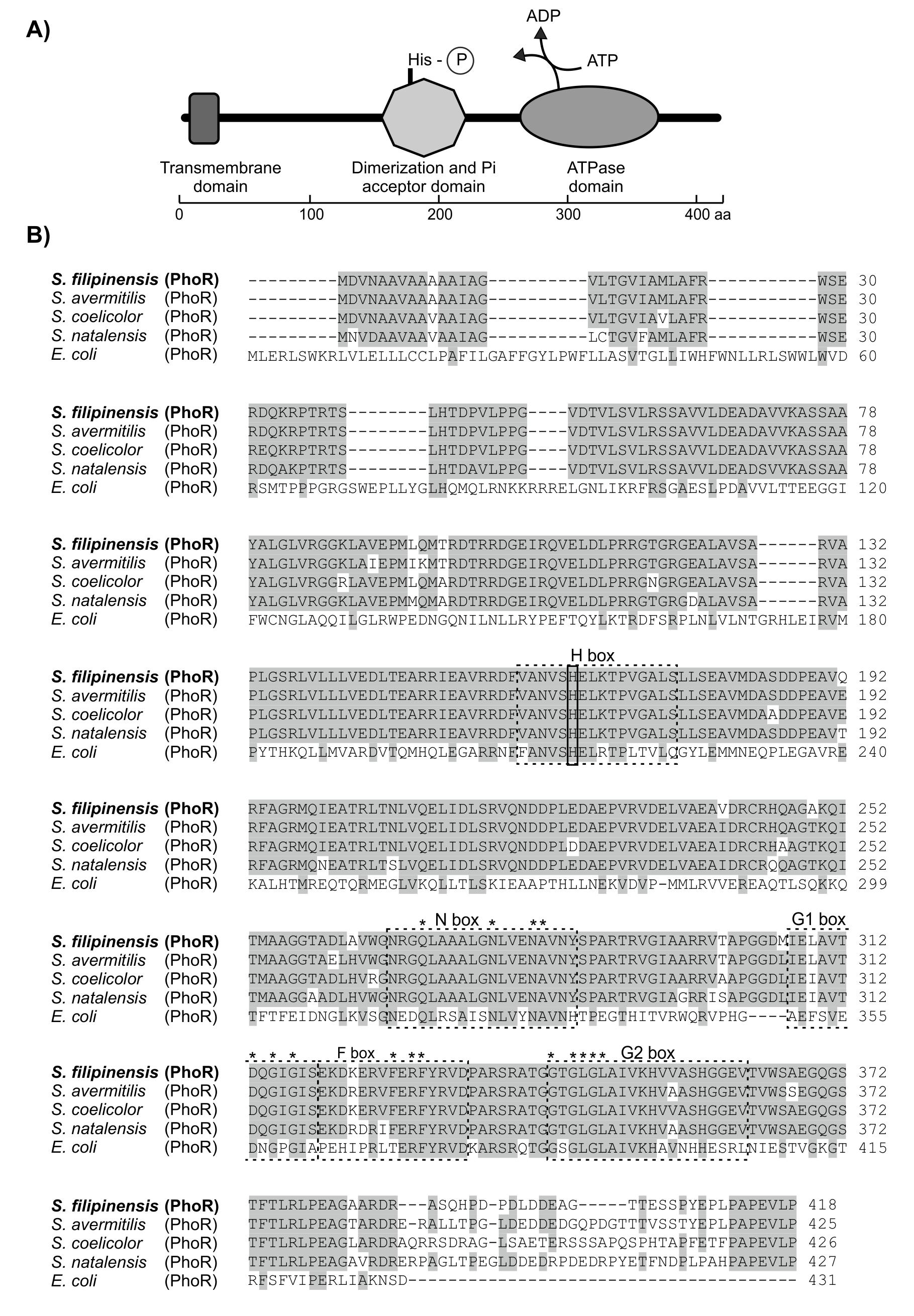

Supplement: S1 Fig — A) Predicted PhoR domains. B) Alignment of S. filipinensis PhoR with its orthologues from S. avermitilis (BAC71685), S. coelicolor (CAB77323), S. natalensis (CAJ45043) and E. coli (P08400). Identical amino acids in at least three of the five sequences are shaded. The amino acid residues that form boxes H, N, G1, F and G2 are framed by a dashed line and the amino acids that make up the conserved motifs are indicated by an asterisk. Histidine that is autophosphorylated (H165 in S. filipinensis) is boxed. (TIF) [file pone.0208278.s001.tif]

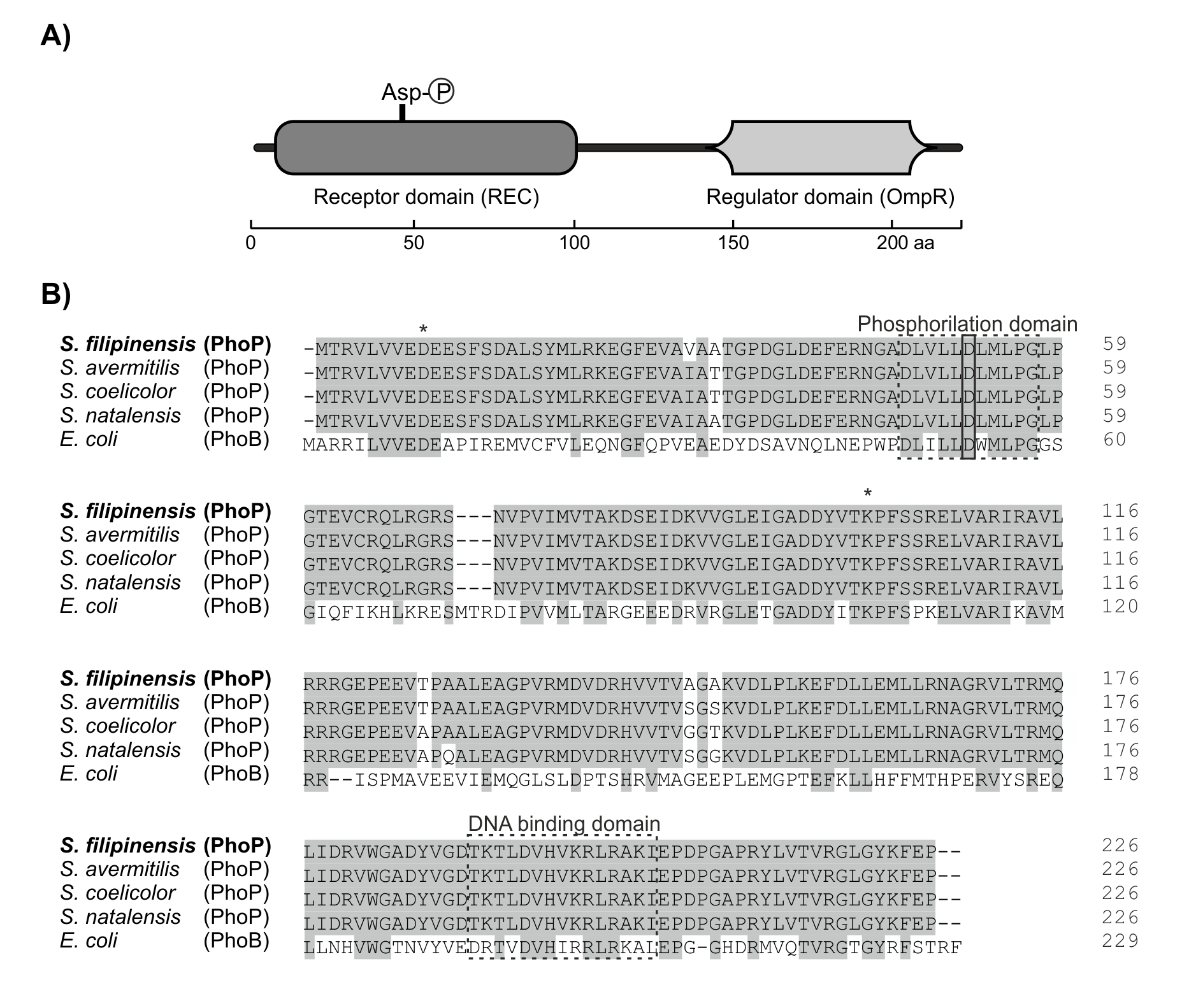

Supplement: S2 Fig — A) Predicted PhoP domains. B) Alignment of PhoP with its orthologues from S. avermitilis (BAC71684), S. coelicolor (CAB77324), S. natalensis (CAJ45043) and E. coli (P0AFJ5). Identical amino acids in at least three of the five sequences are shaded. The amino acid residues that make up the phosphorylation domain and the DNA binding domain are framed by a dashed line and the amino acids important for phosphorylation are indicated by an asterisk. The aspartic residue phosphorylated by PhoR (D52) is boxed. (TIF) [file pone.0208278.s002.tif]

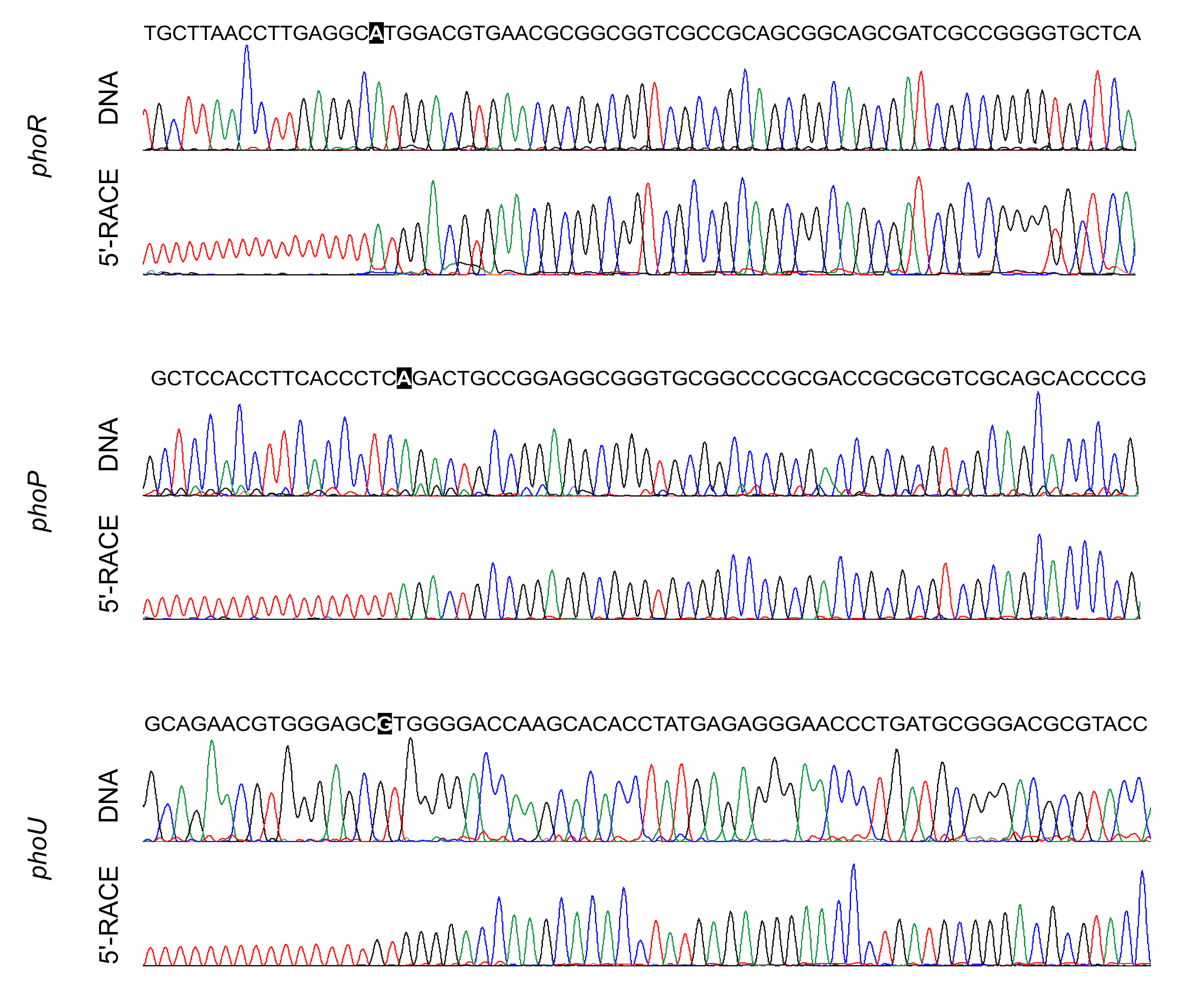

Supplement: S3 Fig — Genomic sequences are indicated at the top, and RACE results at the bottom. (TIF) [file pone.0208278.s003.tif]

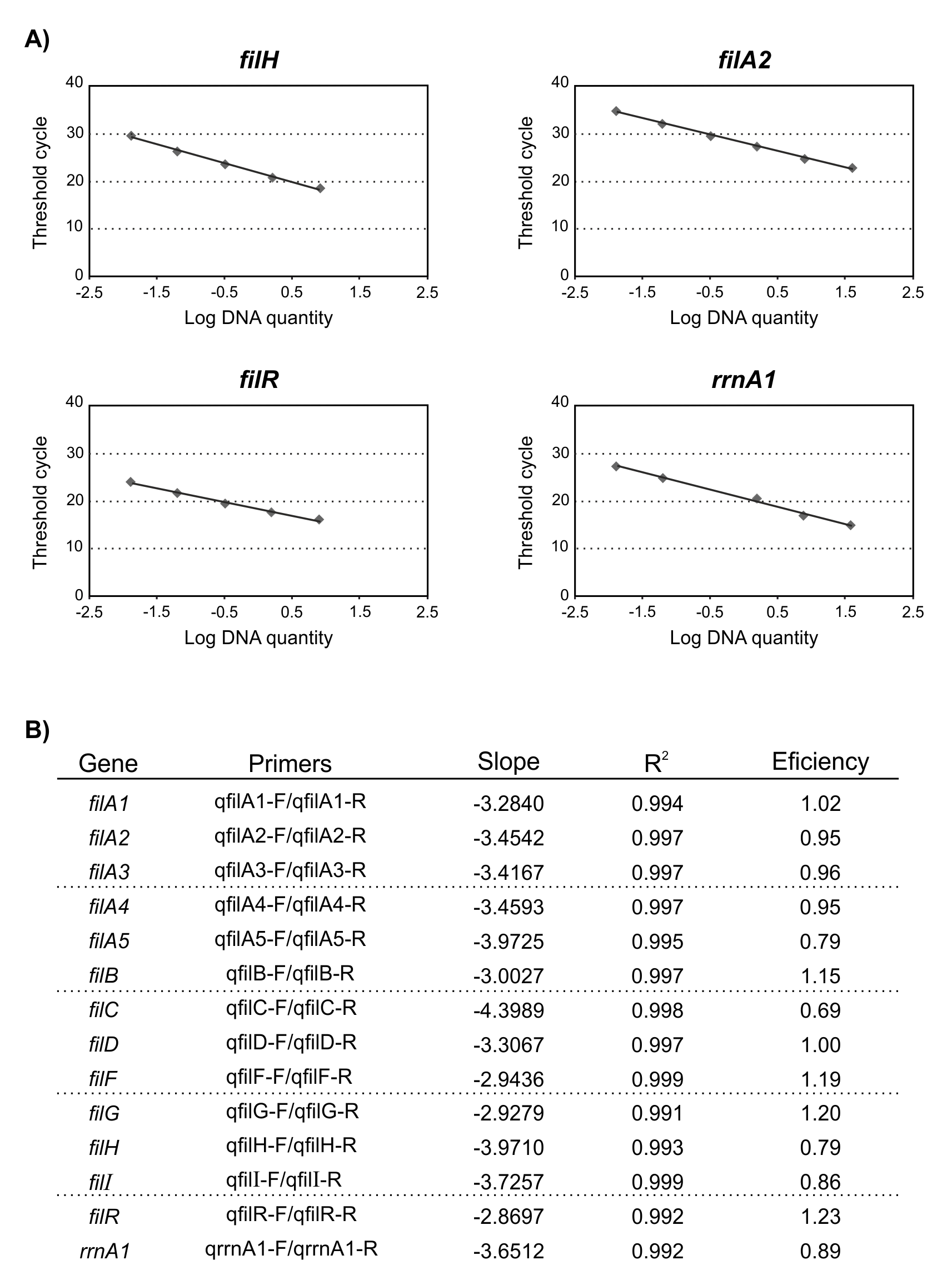

Supplement: S4 Fig — The efficiency of each set of primers was calculated according to the equation E = 10[-1/slope]-1. Using 5-fold dilutions of genomic DNA, the resulting Ct values were plotted against the logarithm of the DNA as shown in A) for filH, filA2, filR and rrnA1. Data are from three replicates, values represent the mean and the vertical bars ± SD. Panel B summarizes information obtained from all plotted data. (TIF) [file pone.0208278.s004.tif]
