# Supplementary material for: Phosphate effect on filipin production and morphological differentiation in Streptomyces filipinensis and the role of the PhoP transcription factor
Source: PLoS One. 2018 Dec 6;13(12):e0208278. doi: 10.1371/journal.pone.0208278 (PMC6283541; doi:10.1371/journal.pone.0208278)
Supplement: S1 Table — (DOCX) [file pone.0208278.s005.docx]

**S1 Table. Primers used for reverse transcription-quantitative PCR.**

| **Name** | **Sequence (5’→3’)** | **Transcripts quantification** | **Product size (bp)** |
| --- | --- | --- | --- |
| qfilA1-F | CGGCTTCCTCGACAGCATC | *filA1* | 114 |
| qfilA1-R | GCTTCCCAGGCCAACTCC |  |  |
| qfilA2-F | CGAGGATCTGTGGGAGTTGGTC | *filA2* | 128 |
| qfilA2-R | CGCGGGCGTAGCTGGTC |  |  |
| qfilA3-F | TGACGAACGAAGACAAGCTC | *filA3* | 126 |
| qfilA3-R | AGCTCATGCCCACGATGG |  |  |
| qfilA4-F | AGGGCGACAGCGACAGC | *filA4* | 138 |
| qfilA4-R | GCGCCACCAGCGAGGAC |  |  |
| qfilA5-F | ACTCCGCCAGTGTGATGTCC | *filA5* | 96 |
| qfilA5-R | CCACGAGCGACGACGAG |  |  |
| qfilB-F | GCCGAAGCAGGCGTTCC | *filB* | 116 |
| qfilB-R | CCAGCGAGGACCACACG |  |  |
| qfilC-F | CCTGCTGCGCGACTCCTC | *filC* | 118 |
| qfilC-R | GTGCTCCGGCTCGTCCTG |  |  |
| qfilD-F | CTTCCCCCTGATCGGTGTC | *filD* | 141 |
| qfilD-R | GCCAGCATGTCGTCCAAGAG |  |  |
| qfilF-F | ATCCAGCAGGCGAACCAG | *filF* | 125 |
| qfilF-R | TTGGAGAATTGACGCACCAG |  |  |
| qfilG-F | GCGGGGCAGACTACGATCAC | *filG* | 100 |
| qfilG-R | CGGTGCCGACCACGAC |  |  |
| qfilH-F | CTCCGCCAGCTTCTACTTCC | *filH* | 147 |
| qfilH-R | AGGGCCTCGTAGATCTTGTC |  |  |
| qfilI-F | CGGTCCATCGGCTACTTCTG | *filI* | 80 |
| qfilI-R | ATCCATCCGTCGTTCTCCATG |  |  |
| qfilR-F | AGACATGGCTCTGGAGTGTG | *filR* | 82 |
| qfilR-R | GTGCCCACCGAACTGCTC |  |  |
| qrrnA1-F | GACGCAACGCGAAGAACC | *rrnA1* | 137 |
| qrrnA1-R | TGCGGGACTTAACCCAACATC |  |  |
