# Supplementary material for: Phosphate effect on filipin production and morphological differentiation in Streptomyces filipinensis and the role of the PhoP transcription factor
Source: PLoS One. 2018 Dec 6;13(12):e0208278. doi: 10.1371/journal.pone.0208278 (PMC6283541; doi:10.1371/journal.pone.0208278)
Supplement: S2 Table — (DOCX) [file pone.0208278.s006.docx]

**S2 Table. Primers used for rapid amplification of cDNA ends.**

| **Name** | **Sequence (5’→3’)** | **TSP** |
| --- | --- | --- |
| PhoPin-RACE-1 | GTCGATCTCGCTGTCCTTGGC | *phoP* |
| PhoPin-RACE-2 | CACCTCCGTACCGGGCAGACC |  |
| PhoPin-RACE-3 | CGGCAGCATCAGGTCGAGGAG |  |
| PhoR-RACE-1 | CCGGGTCATCTGGAGCATCG | *phoR* |
| PhoR-RACE-2 | GCGGAGCACGGACAGTACGG |  |
| PhoR-RACE-3 | GGTCGGTCGCTTCTGGTCGC |  |
| PhoU-RACE-1 | GGATGGTCGCGTGCAGGTC | *phoU* |
| PhoU-RACE-2 | CGTGCTGGGCGAGGTCGC |  |
| PhoU-RACE-3 | CGCAGGGAGGTGACGACGATAC |  |
