# Supplementary material for: Phosphate effect on filipin production and morphological differentiation in Streptomyces filipinensis and the role of the PhoP transcription factor
Source: PLoS One. 2018 Dec 6;13(12):e0208278. doi: 10.1371/journal.pone.0208278 (PMC6283541; doi:10.1371/journal.pone.0208278)
Supplement: S3 Table — (DOCX) [file pone.0208278.s007.docx]

**S3 Table. Primers used for probe amplification for EMSAS.**

| **Name** | **Sequence (5’→3’)** | **Promoter** | **Product size (bp)** |
| --- | --- | --- | --- |
| PhoU1F | CTCCACCAGACCGTCGCCG | *phoU-RP* | 298 |
| PhoR6R | CGATGACGCCGGTGAGCAC |  |  |
| PhoR4F | GCACGGCGGGGAGGTCAC | *phoP* | 351 |
| PhoP6R | CGGCAGCATCAGGTCGAGGAG |  |  |
| FilA1pF | GCGGAGATCATCTTGGTGGCG | *filA1* | 336 |
| FilA1pR | GCACTCCACCCACCCACACAG |  |  |
| FilA2pF | GCTCGGCAAGGAATTCGGG | *filA2* | 385 |
| FilA2pR | CCTCGCGGGCGTAGCTGG |  |  |
| FilA3pF | CGGCGAGCGACGAGGAGC | *filA3* | 540 |
| FilA3pR | CAGCGACTGGACGGCCAGGTGC |  |  |
| FilRFpF | GAGGTGTGGGCATGGTGGTCC | *filR-F* | 550 |
| FilRFpR | GGCTGCTGCACGCTCGGG |  |  |
| FilGpF | GGACGACTTCGTGAAGTGAGCGG | *filG* | 513 |
| FilGpR | CGAACGTGCCGCCGGAACC |  |  |
